# Naïve chicks do not prefer objects with stable body orientation, though they may prefer behavioural variability

**DOI:** 10.1007/s10071-023-01764-3

**Published:** 2023-03-18

**Authors:** Orsola Rosa-Salva, Mikołaj Hernik, Martina Fabbroni, Elena Lorenzi, Giorgio Vallortigara

**Affiliations:** 1 CIMeC Center for Mind/Brain Sciences, University of Trento, Rovereto, TN Italy; 2 UiT The Arctic University of Norway, Tromsø, Norway

**Keywords:** Domestic chicks, Inborn social predispositions, Animacy, Agency, Body orientation, Variability

## Abstract

Domestic chicks (*Gallus gallus domesticus*) have been widely used as a model to study the motion cues that allow visually naïve organisms to detect animate agents shortly after hatching/birth. Our previous work has shown that chicks prefer to approach agents whose main body axis and motion direction are aligned (a feature typical of creatures whose motion is constrained by a bilaterally symmetric body plan). However, it has never been investigated whether chicks are also sensitive to the fact that an agent maintains a stable front–back body orientation in motion (i.e. consistency in which end is leading and which trailing). This is another feature typical of *bilateria,* which is also associated with the detection of animate agents in humans. The aim of the present study was to fill this gap. Contrary to our initial expectations, after testing 300 chicks across 3 experimental conditions, we found a recurrent preference for the agent which did not maintain a stable front–back body orientation. Since this preference was limited to female chicks, the results are discussed also in relation to sex differences in the social behaviour of this model. Overall, we show for the first time that chicks can discriminate agents based on the stability of their front–back orientation. The unexpected direction of the effect could reflect a preference for agents’ whose behaviour is less predictable. Chicks may prefer agents with greater behavioural variability, a trait which has been associated with animate agents, or have a tendency to explore agents performing “odd behaviours”.

**Supplementary Information:**

The online version contains supplementary material available at 10.1007/s10071-023-01764-3.

## Introduction

Predispositions to approach or attend to stimuli resembling animate creatures (henceforth “social predispositions”) are one of the most intriguing aspects of early social development. For instance, similar social predispositions towards face-like stimuli have been observed in human newborns (Morton and Johnson 1991; Buiatti et al. 2019), non-human primates (Sugita 2008) and visually naïve domestic chicks (Rosa-Salva et al. 2010, 2011, 2012). These predispositions can have an important adaptive role, especially in social species. Social predispositions allow young organisms to establish an early contact with potential caregivers. At the same time, they also stimulate the developing nervous system to specialise circuits devoted to sophisticated processing of socially relevant information (Johnson 2005). This may be particularly important for nidifugous bird species, precocial animals that leave the nest shortly after hatching, such as domestic chicks. Nidifugous species are characterised by prominent filial imprinting, which restricts their social attachment to the first conspicuous objects they are exposed to (McCabe 2013). It is, thus, of paramount importance for chicks to direct their attention towards appropriate imprinting objects, such as siblings or the mother hen, from the very first hours after hatching. Numerous studies have investigated social predisposition manipulating the potential imprinting objects available to visually naïve chicks and testing their spontaneous preference between them (reviewed in Di Giorgio et al. 2017; Rosa-Salva et al. 2021). This has made domestic chicks one of the most popular models for the study of social predispositions.

Recent studies have focused on the role of dynamic information in directing social predispositions. Newborn babies and chicks alike are attracted by objects whose motion resembles that of animate agents. Self-propulsion is one of the key features that characterise animate motion. Animate agents possess an internal energy source, empowering them to move independently from the action of external forces. Among the cues of self-propulsion, we know that social predispositions are elicited by the capacity of an agent to start moving from a resting state (chicks: Mascalzoni et al. 2010; newborn babies: Di Giorgio et al. 2016), spontaneous speed changes (chicks: Rosa-Salva et al. 2016; babies: Di Giorgio et al. 2021) and by rotational movements (Rosa-Salva et al. 2018).

Self-propulsion, however, is not the only distinctive characteristic of animate motion. The body structure of animate agents typically constrains the ways in which they can move. This causes agents to move in recognisable ways, which can be exploited by the visual system to quickly detect potential social companions. For instance, both human newborns and naïve chicks show social predispositions for the semi-rigid movement caused by the skeletal structure of most legged creatures (Vallortigara et al. 2005; Simion et al. 2008). Chicks also show a preference for aligning with the motion direction of a point light display (PLD) of a walking hen (Vallortigara and Regolin 2006). Moreover, in both species, the preference for / tendency to align with the semi-rigid motion is restricted to canonical vertical orientation of the stimuli and goes away, if the same semi-rigid motion is presented upside down (Vallortigara and Regolin 2006; Simion et al. 2008; Bardi et al. 2011, 2014).

The bilaterally symmetrical body with its anteroposterior organisation, which characterise all of *bilateria*, also constrains their motion. *Bilateria* tend to align their main body axis with their motion direction and tend to move facing forwards. Human observers give higher animacy ratings to agents whose main axis is parallel to the motion trajectory (Tremoulet and Feldman 2000). The human visual system seems also to implicitly “expect” symmetrical objects to maintain this parallelism and directed objects to face forward, when moving (McBeath et al. 1992; Morikawa 1999; Pavlova et al. 2002; Dolgov et al. 2009; Jardine and Seiffert 2011). Likewise, human infants expect the constraints of bilaterian morphology on behaviour to be stable over time. After observing a novel bilaterian agent act, 6 and 7 months-old expect it to maintain the same front–back orientation with respect to motion direction in future actions (Hernik et al. 2014; Wronski 2022).

Similarly, visually naïve domestic chicks display a preference for agents characterised by parallelism between the main body axis and the motion path. In fact, chicks consistently prefer to approach an animated elongated shape, which moves along its main axis and rotates by 90 degrees when changing motion direction by 90 degrees (Rosa-Salva et al. 2018). This behaviour realigns the agent’s axis to the changed trajectory, maintaining parallelism and keeping the same end forwards. In a critical test, chicks preferred this stimulus over a closely matched distractor. In the distractor stimulus, an identical shape also realigned to the 90-degree change in the trajectory by rotating 90 degrees, and it kept the same end forward, but its main axis was always “tilted” rather than parallel with respect to its motion path (Rosa-Salva et al. 2018, experiment 2).

It remains unknown, however, whether merely maintaining stable front–back orientation in motion (or more broadly: exhibiting consistency in which end is leading, and which trailing) can elicit preferential approaches in chicks. Our earlier study suggests that consistency of front–back orientation is at least not as effective in attracting chick’s attention as axis-path parallelism and rotational movement are (Rosa-Salva et al. 2018, experiments 4–5). Indeed, in our previous study (Rosa-Salva et al. 2018) we did not find evidence for a role of the consistency of front–back orientation in directing chicks’ preferences, when this factor was dissociated from the presence of parallelism and rotational movements. For instance, all other factors being equal, chicks showed a preference for a stimulus showing a wider rotational movement over a stimulus showing consistency of front–back orientation. However, none of the experiments we performed in Rosa-Salva et al. (2018) were designed to directly address this question, nor allowed us to test the role of axis-path parallelism in the processing of front–back consistency. The aim of the current study was, thus, to test directly for the role of front–back consistency (i.e. of keeping the same end as the leading end, and the same end as the trailing end) in chicks’ social preference. To do so, we compared chicks’ preferences for one target stimulus showing front–back consistency against a distractor stimulus matched to the target on numerous properties, yet not showing the front–back consistency. To a human observer, the target stimulus could appear as always moving forwards (or always moving backwards) and the distractor stimulus could appear to alternate between moving forwards and backwards.

The design also involved the following key elements. (i) To study the role of axis-path parallelism in eliciting preferences for front–back consistency, we assessed chicks’ choices for a target stimulus vs. a distractor stimulus under two conditions: when both stimuli kept their main axes parallel to motion path vs. when neither stimulus showed this parallelism. (ii) To minimise competition with other known motion cues of animacy, the amount of motion (including the rotational motion) in the target and the distractor movies was identical. (iii) To facilitate the tracking of front–back consistency (and the detection of inconsistency), one end of the stimulus was marked with a distinctive abstract feature, while the other was plain. Finally, since early social behaviour and social motivation can differ between male and female chicks (e.g. Workman and Andrew 1989; Vallortigara and Andrew 1991; Vallortigara 1992; Regolin et al. 2000; Miura and Matsushima 2012; Versace et al. 2017), preferences were also compared between male and female individuals.

Point (i) is of particular significance, since most *bilateria* (including ancestral vertebrates) align their body axis with the direction of locomotion. It is, therefore, conceivable that detecting parallelism between the axis and the motion path would be one of the input conditions for the processing of other motion properties associated with the constraints of bilateral symmetry on movement. It was, thus, important to test whether the presence vs. absence of the alignment between the stimulus’ main axis and its motion trajectory modulates the predicted preference for front–back consistency.

## Material and methods

### Subjects and hatching conditions

For each condition, we used 100 visually naïve domestic chicks (*Gallus gallus domesticus*) of the Aviagen Ross 308 strain. The sample size, which was decided a priori and set on a round number for practical reasons, is in the range indicated by power analyses based on Rosa-Salva et al. 2018. For the four experiments reporting statistically significant effects in Rosa-Salva et al. (2018), effect size, Cohen’s *d*, ranged from 0.241 to 0.298 (average effect size *d* = 0.278). Based on these data, the required sample size (calculated for a one-sample two-tailed *t* test, and 1-*ß* = 0.8) ranged from 92 to 138, while the required sample size calculated on the basis of the average effect size was 104 chicks.

Chicks hatched in the lab from fertilised eggs, provided by a commercial hatchery (CRESCENTI Società Agricola S.r.l. –Allevamento Trepola– cod. Allevamento127BS105/2, Italy). Until day 19 of incubation, eggs were kept in a FIEM-MG 200/300 Super Rural incubator (temperature 37.7 ℃; humidity 40%). Afterwards, the eggs were transferred to a hatchery (FIEM, MG 140/200 rurale, 37.7 ℃, 60% humidity). The incubator, the hatchery and the hatching room were kept in complete darkness. On the hatching day, until the testing, chicks stayed in the incubator in complete darkness at a temperature of 33 ℃. After the testing, chicks were donated to local farmers.

### Procedure and apparatus

The general procedure and apparatus were the same as in our previous studies (e.g. Rosa-Salva et al. 2016, 2018).

#### Apparatus

The test apparatus was a white, longitudinal corridor (85 × 30 × 30 cm) closed at the two ends by computer monitors (LCD Monitor BenQ XL2410T, 120 Hz) displaying the experimental stimuli (60 frames/s). At each end of the apparatus, a 30 × 30 cm portion of the monitor (henceforth: the scene) was visible to the chicks. The corridor was divided into the central sector (45 × 30 cm) and two identical lateral sectors (20 × 30 cm each), which were adjacent to the two monitors and elevated 1.5 cm above the central sector. The animal had to climb up this small step when entering any of the two lateral sectors to approach the stimuli. The apparatus was illuminated only by the two stimulus monitors.

#### Procedure

Each chick was tested only once on the first day after hatching. The animal was taken from the incubator in complete darkness and carried in a closed opaque container to the experimental room. To begin the test, the chick was placed in the centre of the apparatus, facing one of the two long walls. The position of the target stimulus in the apparatus and the initial orientation of the chicks towards one long wall or the other was counterbalanced between subjects in each experiment. A video camera recorded the testing from above the corridor and fed the image to the screen located in the same room, on which the experimenter observed the chick’s behaviour and coded it online. Chicks’ behaviour was coded online with a purpose-built MATLAB script. The software calculated the time (in seconds) spent by the chick in each sector during the test, based on the experimenter’s button presses indicating entering any of the three sectors. Two dependent measures have been analysed for this study: (i) the first stimulus approached by each subject (i.e. the first lateral sector entered during the test); (ii) the percentage of time spent (on the platform) near the target stimulus over the total choice time, calculated as:

Time spent near the target stimulus/(time spent near the target stimulus + time spent near the distractor stimulus) × 100.

Values of this index could range from 0% (full preference for the distractor stimulus), to 100% (full preference for the target stimulus), whereas 50% represented the absence of preference.

Whenever the behaviour of the chick was considered ambiguous, test was re-coded offline by a second coder, blinded to the stimuli position and to the sex of the chick. In this case, the offline blind coding only was used for the analyses.

### Stimuli

**Fig. 1:**
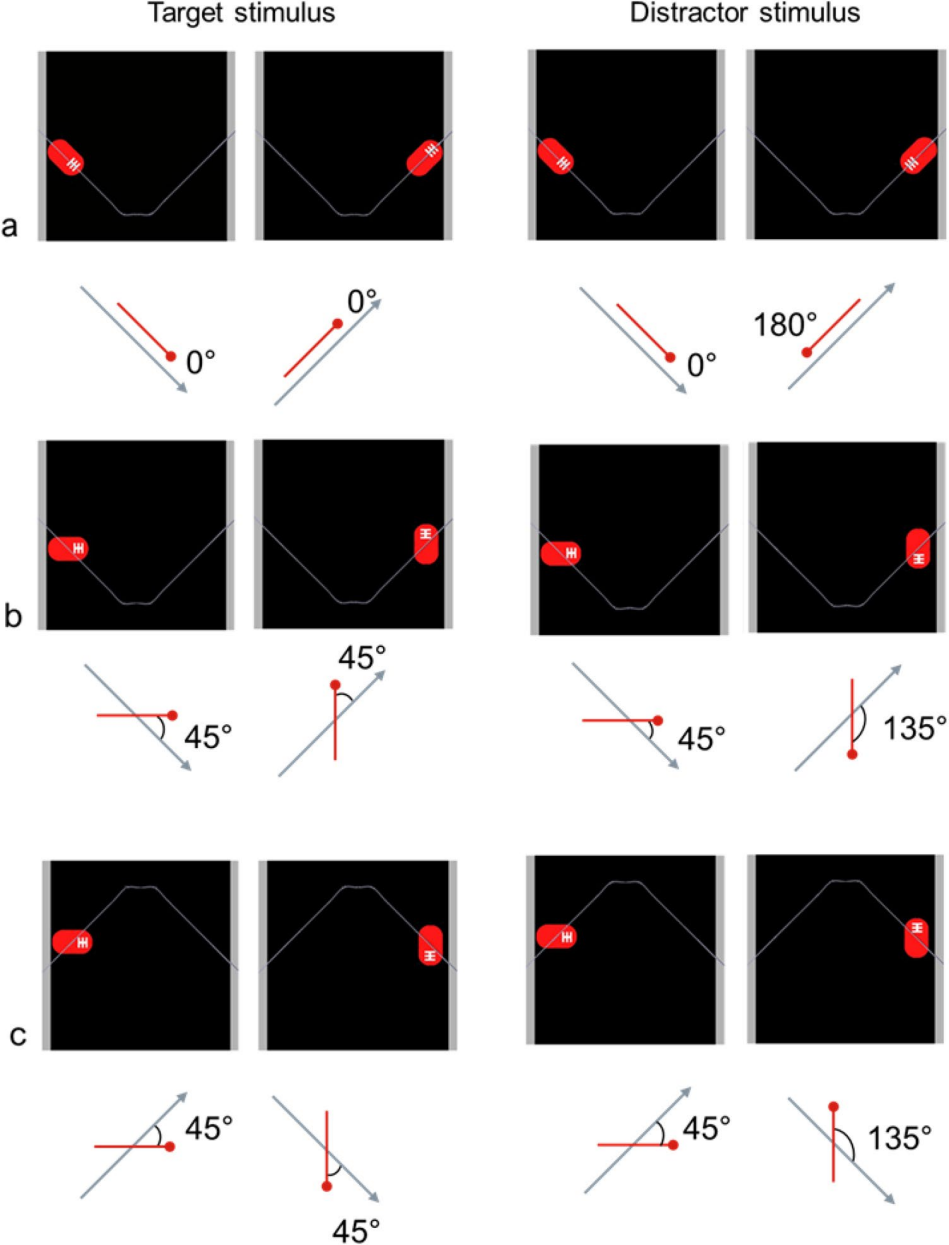
Schematic representation of the stimuli of the parallel (**a**), the non-parallel condition of Experiment 1 (**b**) and 2 (**c**). For each stimulus, two frames are shown to illustrate the orientation of the agent in the first and second part of its trajectory (the frames are taken from the first half of the animation, in which the agent moves rightwards). The grey line (not visible to the chicks) illustrates the trajectory followed by the centre point of the agent. Below each frame, a drawing illustrates the angle created by the agent’s body axis (in red) and the trajectory (grey arrow). In this drawing, a dot has been added to represent the end of the agent marked with the white feature. Note that the absolute amount of rotational motion of the agent is identical in all the movies and equals to 90 degrees

Stimuli were animations created in Adobe Aftereffects (CS6 version 11.0.2) software. The general structure of the stimuli was similar to that used in Rosa-Salva et al. (2018). Each stimulus displayed a red rectangular shape (6.3 × 3.7 cm) with rounded corners (henceforth: the agent). Unlike in earlier studies, there was a white geometrical feature (inscribed in a rectangle of 1.6 × 1.6 cm, composed of 0.3 cm thick stripes) on one of the agent’s extremities (the leading extremity when the agent entered the scene). The agent moved on a symmetrical U-shaped path at a constant speed over a uniform black background. The background was delineated by two grey vertical edges (each 1.25 cm wide) inserted digitally on each side of the scene. The scene itself was delimited by the lateral walls of the apparatus. The start and the end points of the agent’s trajectory were occluded. Thus, the agent entered and exited the scene in motion, emerging from behind the edge on one side and slipping behind one on the opposite side. Each motion cycle begun with the agent entering the scene in its upper-left part. The agent then moved towards the mid-point at the bottom of the scene, then upwards again and went out of view in the upper-right part of the scene (Fig. 1). Soon after disappearing from view, the agent re-emerged from where it had exited on the right side and took the same U-shaped path leftwards. The cycle ended with the agent exiting at the upper-left side. Each cycle lasted 12 s. Each test trial lasted for 6 min, during which the cycles were looped seamlessly.

In all stimuli for Experiment 1, at the point of trajectory change (the “dip” of the U-shaped path), the agent rotated by the same absolute number of degrees, 90°. However, the rotation direction differed between the target and the distractor stimuli. In the target stimuli, the agent rotated counter-clockwise when moving rightwards and clockwise when moving leftwards. In the distractor stimuli, the agent rotated clockwise when moving rightwards and counter-clockwise, when moving leftwards. Stimuli for Experiment 2 were constructed the same way but presented upside down. Overall, in all the target stimuli, the agent maintained front–back consistency throughout: the same marked end was leading and the same plain end was trailing before and after the change of trajectory. In contrast, in the distractor stimulus, the agent always moved with its marked end leading (and the plain end trailing) during half of its trajectory and with the plain end leading (and the marked end trailing) in the other part of its trajectory. Thus, in all the distractor stimuli, the agent’s motion lacked front–back consistency.

### Analyses

The frequency of first approach towards the two stimuli was compared with a Chi-square test of independence. The distribution of frequencies across sexes and experimental conditions was analysed applying Chi-square test on a contingency table or with a log-linear regression, when it was needed to analyse the interaction of more than two factors.

The percentage of time spent near the target stimulus was compared to the value of 50% (chance level, representing no preference between the stimuli) using a one-sample two-tailed *t *test. Independent-sample *t *tests or univariate multifactorial ANOVA were used to compare the percentage of time spent near the target stimulus by male and female chicks and/or in different experimental conditions.

All statistical analyses were performed with the IBM software SPSS (version 26), except for the log-linear regression and meta-analytical estimates (JASP software; JASP Team 2019) and power analyses (G*Power, version 3.1.9.4, Erdfelder et al. 1996).

## Experiment 1

The aim of this experiment was twofold. We wanted to test the hypothesis that front–back consistency alone can elicit social predisposition in visually naïve chicks. Moreover, to investigate the role of axis-path parallelism in this process, we tested chicks’ preferences under two conditions. In the parallel motion condition, the agent in both stimuli kept its main axis parallel to the motion path. In the non-parallel motion condition, the agent in neither stimulus showed this parallelism, but rather its main axis was tilted with respect to the motion path.

With regard to the first aim, we expected a preference for the agent behaving consistently (target stimulus). With regard to the second aim, we hypothesised that the expected preference for the agent showing consistency of the front–back orientation may be limited to, or show a stronger effect in, the parallel motion condition.

### Subjects

*N* = 200 visually naïve chicks (100 for each condition, of which 54 males in the parallel condition and 52 males in the non-parallel condition).

### Stimuli

Parallel condition: The target agent maintains an angle of 0° between its main symmetry axis and its trajectory, keeping its marked end as the leading end and thus exhibits consistency of front–back orientation. The distractor alternates between 0° and 180°, with its plain and marked extremity alternating as the leading end (lack of front–back consistency). Parallelism is present for both agents (Fig. 1a and supplementary materials S1).

Non-parallel condition: The target agent maintains an angle of 45° between its main symmetry axis and its trajectory. The target agent, thus, keeps the same end (marked by the white feature) as the leading end, displaying consistency of front–back orientation. The distractor alternates between 45° and 135°, in the two halves of its trajectory. Thus, it alternates between leading with its marked or its plain end, lacking front–back consistency. Parallelism is absent for both agents. (Fig. 1b, see also supplementary materials S2).

### Results

**Fig. 2:**
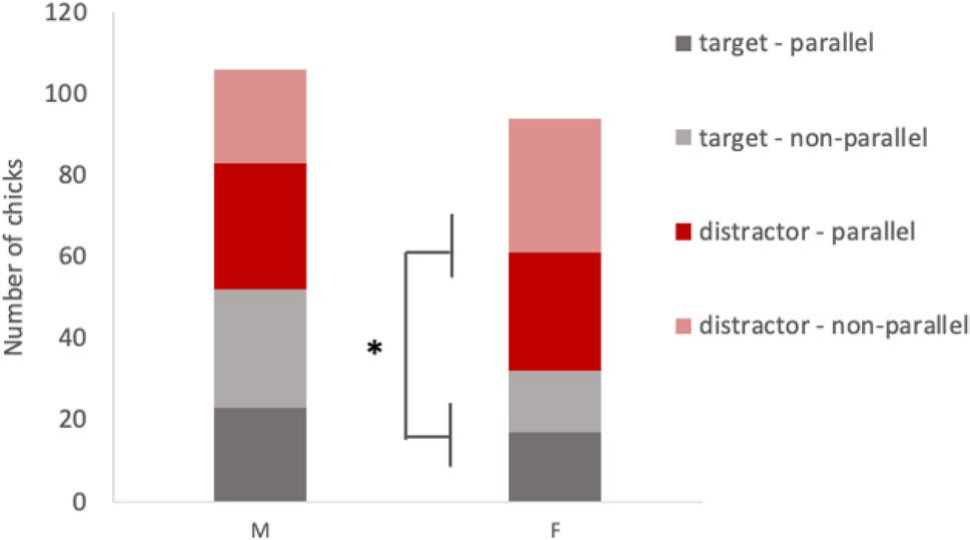
Results of Experiment 1 for the dependent variable first stimulus approached. The number of chicks is represented on the Y-axis. Approaches to the target and distractor stimulus are represented in grey and red, respectively. Data from the parallel and the non-parallel condition are represented in darker and lighter shades respectively, while data from male (M) and females (F) are represented in two columns. Asterisks indicate significant differences (* <0.05)

A log-linear regression was performed to study the association of *first approach* (target vs. distractor), *condition* (parallel condition vs. non-parallel condition) and *sex* (male vs. female) (Fig. 2). This revealed that including the term condition did not improve fit of the model. The model including main effects of sex, first approach and their interactions revealed sex (*Z* = 2.16, *p* = 0.03) as a significant factor and significant sex by approach interaction (*Z* = 2.14, *p* = 0.03). While males did not approach any of the two stimuli above chance level (52 chicks approached the target first and 54 approached the distractor first *X*^2^_1_ = 0.038, *p* = 0.923), females approached significantly more often the distractor (32 approached first the target and 62 approached the distractor first, *X*^2^_1_ = 9.574, *p* = 0.003).

**Fig. 3:**
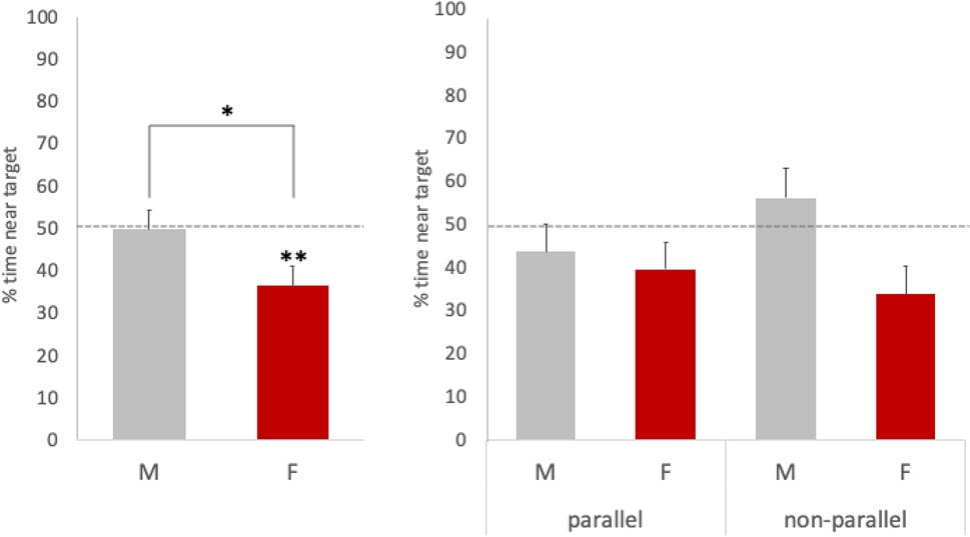
Results of Experiment 1 for the percentage of time spent near the target stimulus over the total choice time. Mean percentage and S.E.M. are plotted. Value for males and females is represented in grey and red, respectively. In the left panel, data are collapsed across the two conditions; in the right panel, the distribution is shown across conditions. Asterisks indicate significant differences (* <0.05, **<0.01). Chance level is represented by the dotted line

A two-way ANOVA (between-subjects factors: *condition* and *sex*) on the percentage of time spent near the target did not reveal any significant difference between the two conditions (*F*_1,196_ = 0.271, *p* = 0.603) nor interaction between the two factors (*F*_1,196_ = 1.928, *p* = 0.197). However, a significant effect of sex emerged from this analysis (*F*_1,196_ = 4.091, *p* = 0.044) (Fig. 3). While males did not exhibit any preference between the two stimuli (*t*_105_ = − 0.041, *p* = 0.968, mean = 49.8%, SEM = 4.6%, C.I. =  − 0.094 to 0.09, *d* = 0.003), females showed a preference for the distractor (*t*_93_ =  − 2.943, *p* = 0.004, mean = 36.6%, SEM = 4.5%, C.I. =  − 0.224 to − 0.043, *d* = 0.303). The size of this effect was smaller in the parallel condition (*d* = 0.241) than in the non-parallel condition (*d* = 0.359).

### Discussion

Contrary to our hypothesis, we did not observe a preference for the agent maintaining stable front–back orientation. Instead, we found a significant preference for the distractor in female, but not in male, chicks (see the General discussion for considerations on sex differences). This result was not predicted. To interpret it, we considered what aspects of the distractor stimuli used in Experiment 1 could attract the chicks. We observed that the 90° rotational motion of the distractor–agent combined with its horizontal displacement shows a similarity to the motion pattern of the lower extremity of a limb. E.g. a foot in gait, where the part of the limb further from the surface is displayed in the direction of global motion of the object, while the part of the limb close to the surface either stays in place or is displaced in the direction opposite to the global direction of motion. This distant similarity could be relevant, because it has been shown that in point-light displays (PLD) local information, especially conveyed by the dots corresponding to the feet of the walking creature, is crucial for biological motion perception. Indeed, feet-dots can convey biomotion perception to human observers also when presented in isolation from the rest of the figure (Chang and Troje 2008, 2009a). In contrast, some indirect evidence seems to indicate that rats are unable to extract motion information from the feet-dots of PLDs (MacKinnon et al. 2010). Other studies, however, suggest that also animal species can process local information provided by single dots of the PLD displays, regardless of the overall configuration (primates: Parron et al. 2007; Vangeneugden et al. 2010; pigeons: Troje and Aust 2013; dogs: Eatherington et al. 2019). For instance, most pigeons seem to prioritise local information from the motion of single dots, over the global information provided by the whole PLD configuration. Moreover, they seem to be particularly affected by the information provided by the feet-dots and by their position relative to the rest of the configuration (Troje and Aust 2013). While in chicks the perception of biological motion from single feet-dots has never been tested, indirect evidence suggests that the motion of single body parts (such as the head) may be particularly relevant in the perception of the biological motion of walking conspecifics (Miura et al. 2020).

Thus, even though our stimuli showed a single rigid shape rather than a semi-rigid PLD, we hypothesised that the mechanism underlying the preference for the distractor in females might have been the same that drives preferences for gait patterns. Experiment 2 was conducted to directly test this post hoc hypothesis. To do so, we exploited another prominent feature of biological motion perception, namely the inversion effect. In humans, biological motion perception from PLD is strongly affected when the stimuli are presented upside down (Troje and Westhoff 2006; Chang and Troje 2009b, a; Hirai et al. 2011b, a). Likewise, in newborn babies, looking preferences for PLD of biological motion are affected by inversion (Simion et al. 2008). Importantly, inversion effects are found also when local information, e.g., from the feet-dots, is presented in isolation (human adults: Troje and Westhoff 2006; Chang and Troje 2008; newborn babies: Bardi et al. 2014). Finally, we know that also chicks lose their spontaneous tendency to align with the apparent movement direction of the PLD of a walking hen, when this is presented upside down (Vallortigara and Regolin 2006). Similar inversion effects have been reported in cats (Blake 1993), dogs (Eatherington et al. 2019) and female marmosets (Brown et al. 2010). Moreover, some of the pigeons tested by Troje and Aust (2013) revealed inversion effects even when relying on the local information provided by the motion of single dots to solve the biological perception task. These inversion effects are considered a trademark of the mechanisms subtending to biological motion perception (e.g. Troje and Westhoff 2006). Thus, if the preference for the distractor was due to its similarity with the motion of a walking foot, presenting the stimuli upside down should have a significant effect on chicks’ behaviour. More specifically, we predicted that if female chicks’ preferences originated from the same mechanisms as biological gait perception, presenting the stimuli upside down should disrupt the attraction for the distractor.

## Experiment 2

The aim of this experiment was to test whether the effect found in female chicks in Experiment 1 will decrease with vertical inversion of the stimuli, consistently with the literature on biological motion perception (Vallortigara and Regolin 2006; Simion et al. 2008; Chang and Troje 2008; Bardi et al. 2014, see above). No similar prediction could be made for males, which did not show any preference between the two stimuli already in the first experiment. Thus, in the current experiment, we focussed mostly on female chicks.

To maximise the effectiveness of our design, here we employed an upside-down version of the stimuli that elicited the strongest preference in Experiment 1, namely those of the non-parallel condition. The behaviour of female chicks presented with those stimuli was compared with that of females from the non-parallel condition of Experiment 1, to reveal whether presenting the stimuli upside down affected female chicks’ performance.

### Subjects

*N* = 100 visually naïve chicks, of which 50 females. Based on the results from the first experiment, we assumed that only females would provide data relevant to our main hypothesis. Females only were, thus, included in the main between-experiment comparison. However, males were included in a further metanalytic comparison of the effect sizes, to provide a fuller and more detailed assessment of the population behaviour.

### Stimuli

Test stimuli were identical to those of the non-parallel condition of Experiment 1, but they were presented upside down (Fig. 1c).

### Results

The behaviour of the female chicks tested in this experiment was compared to the females from the non-parallel condition of Experiment 1 (see the supplementary materials S3 for the full datasets of all experiments, including from male subjects).

In Experiment 2, 23 females approached the target first and 27 the distractor. On average, females spent 40% (SEM = 6%) of their choice time near the target stimulus. A Chi-square test of independence did not reveal any significant difference in the frequency of first approach to the distractor vs. the target stimuli presented in the original orientation (Experiment 1) or upside down (Experiment 2) (*X*^2^ = 0.151, *p* = 0.098, Fig. 3). No significant differences between the two experiments could be detected in the percentage of time spent near the target stimulus either (*t*_96_ = − 0.692, *p* = 0.490, C.I. = − 0.238 to 0.115, *d* = 0.140).

### Meta-analytic comparison

**Fig. 4:**
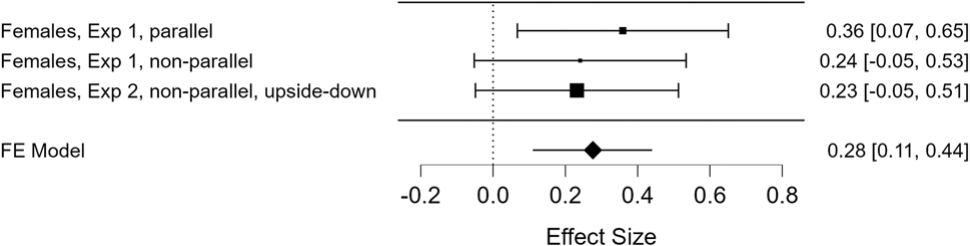
Estimates of effect sizes with confidence intervals, as well as their meta-analytically combined estimate with 95% confidence interval, for female chicks (dependent variable: percentage of time spent next to the distractor stimulus over total choice time)

**Fig. 5:**
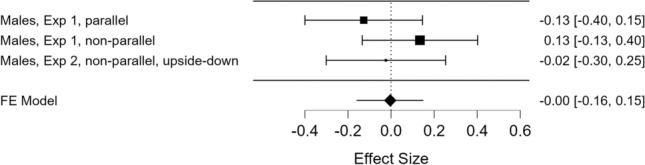
Estimates of effect sizes with confidence intervals, as well as their meta-analytically combined estimate with 95% confidence interval, for male chicks (dependent variable: percentage of time spent next to the distractor stimulus over total choice time)

Finally, to gain a full picture of the behaviour of the two sexes in the conditions run so far, we conducted a final meta-analytic assessment on the percentage of time spent next to the *distractor* stimulus, recorded from both sexes across all the three conditions. It revealed that sex was a statistically significant factor, *Z* = 2.39, *p* = 0.02, Wald test. Two analyses for the two sexes separately revealed a small statistically significant effect for females, *d*_FE_ = 0.28, CI95% [0.11 0.44], *z* = 3.25, *p* = 0.001, and none for males, *d*_FE_ = 0.00, CI95% [− 0.16 0.15], Z =  − 0.05, *p* = 0.96 (Figs. 4 and 5, for females and males, respectively).

### Discussion

Contrary to our post hoc hypothesis, we did not find any significant difference between the behaviour of female chicks tested in Experiment 2, compared to those tested in the non-parallel condition of Experiment 1. Thus, we found no evidence in support of the prediction that an inversion effect should be observed when stimuli are presented upside down. Inversion effects are quite frequent in the perception of biological motion in humans and non-human animals (e.g. Bardi et al. 2014; Blake 1993; Brown et al. 2010; Chang and Troje 2008; Eatherington et al. 2019; Simion et al. 2008; Troje and Aust 2013; Vallortigara and Regolin 2006). While non-significant results should be always interpreted with caution, this points against the post hoc account of the results obtained in Experiment 1, which assumed involvement of mechanisms related to biological motion processing. Alternative explanations for the preferences displayed by female chicks, across all testing conditions, for the distractor stimulus, are presented in the General discussion.

## General discussion

Contrary to our initial hypothesis, Experiment 1 revealed a significant preference for the stimulus presenting inconsistent front–back orientation, limited to female subjects. A similar effect, albeit weak, was found in Experiment 2, in which female subjects were tested with upside-down stimuli. No significant difference could be detected between the behaviour of female chicks tested with non-parallel stimuli in the two experiments. In line with that, the meta-analysis of the effects observed across the three conditions showed statistically significant preference for the distractor stimulus in female chicks and lack of thereof in male chicks. Even though chicks’ preferences were characterised by a small effect size, this pattern seems to emerge consistently across the different experimental conditions.

Stability of body orientation is a feature that can be highly diagnostic of animate intentional agents. In the past, domestic chicks have shown to be sensitive to similar traits associated with the motion of animate agents (Mascalzoni et al. 2010; Rosa-Salva et al. 2016, 2018). Despite that, here we observed a preference for the stimulus lacking a feature hypothesised to be potentially diagnostic of bilateria’s movement. This is particularly surprising, since the design and general procedure of this study are identical to previous works, which successfully demonstrated a preference for other animate/agentive traits (Rosa-Salva et al. 2016, 2018). However, as we mentioned above, our previous studies suggested that the stability of front–back orientation might be less salient for chicks than other properties, such as rotational movement and axis-path parallelism (e.g. Rosa-Salva et al. 2018, Experiments 4–5).

Nevertheless, based on the data we presented, it is difficult to assume that chicks are simply not sensitive to the consistency of front–back orientation. We have observed a preference for non-consistent agents. Thus, we must either identify another property responsible for chicks’ preferences or assume that chicks can discriminate between objects that do exhibit front–back consistency and those that do not, while showing an unexpected preference for the latter. Our *ad interim* hypothesis, linking chicks’ preferences for the distractor to mechanisms related to biological motion processing, was indeed an attempt to follow the first option. However, the lack of any clear inversion effect emerging from the comparison of Experiments 1 and 2, makes this particular account unlikely. It is of course difficult to completely exclude that some other factor, unrelated to both our original experimental question and to biological motion perception, made the distractor more attractive for chicks. However, the two test stimuli were matched in both their static and dynamic features (e.g. trajectory, speed, amount of rotational movements and general visual appearance). We are, thus, currently unable to identify any other confounding factor that could have driven chicks’ preferences. One could also speculate that the extremely simplified nature of the stimuli could have prevented the full expression of some social predispositions (e.g. a preference for gait-like movement). However, in several previous studies, we have employed a similar approach, simplifying the stimuli to isolate the role of single motion features that successfully elicited chicks' social predispositions (e.g. Mascalzoni et al. 2010; Rosa-Salva et al. 2016; 2018; Lemaire et al. 2022).

As a consequence, we must explore the last remaining option, namely that chicks’ behaviour could indeed reflect a sensitivity to the stability of body orientation. In this case, we would interpret our results as a true preference for the stimulus lacking front–back consistency in motion, even though this preference appears weak and detected only in females. There are two orders of concerns with this interpretation. First, we need to ask whether we are measuring a real phenomenon, since in our previous studies we typically observed a preference for the stimuli showing, rather than lacking, the hypothetical animacy/agency cues (e.g. Mascalzoni et al. 2010; Rosa-Salva et al. 2016, 2018). Are we observing merely random variation around the chance level? While the sample of three groups is too small to rule that possibility out, it is notable that the similar unpredicted preference for the distractor was shown by female chicks across all three experimental conditions, whose sample size we determined a priori. We are, thus, concluding at this point that we measured a real effect, even though not a strong one.

The second concern that we need to address is how can we integrate chicks’ preference for the non-consistent stimulus into the standard theory on the adaptive function of chicks’ social predispositions. It is generally assumed that social predispositions should attract chicks to objects that present animacy traits, to direct imprinting towards appropriate social companions. One possibility is that the distractor stimulus used here may actually present more prominent animacy or agency features than the target stimulus, from the chicks’ perspective. For instance, the distractors present greater variability in their behaviour. By necessity, an agent that maintains a consistent body orientation over time displays lower behavioural variability, compared to an agent with an unstable body orientation. Behavioural variability could, in principle, be a cue of animacy or agency. For instance, variability in the route used to approach a goal object is one of the cues that prompt preverbal infants to attribute goal-directed action to unfamiliar moving objects (Biro and Leslie 2007; Csibra 2008). However, this interpretation is at odds with the results of the first experiment of Rosa-Salva et al. (2018). In that case, chicks preferred an agent maintaining parallelism for the whole duration of its movement, over a more variable agent that kept parallelism for only half of its trajectory. Thus, to explain our current results as a preference for variability, we would need to postulate a complex interaction between variability and other cues. To do so, we would need to hypothesise a hierarchy of cues, according to which parallelism, but not stability of body orientation, takes precedence over behavioural variability. According to this model, chicks would prefer the greater amount of parallelism over the greater variability. But, if parallelism is matched between the stimuli, a preference for variability would emerge. Currently, we cannot prove or disprove this hypothesis. However, note that in our most recent experiments (Lemaire et al. 2022), using stimuli that do not possess a main symmetry axis (disks), we found a preference for agents whose reaction to each other’s behaviour was unpredictable. Intriguingly, this sort of stimulus was preferred over agents engaged in social aggregation behaviour and a form of chasing, which should be traits strongly associated with animate agents.

At a very speculative level, we could propose yet another explanation of our results. We could assume that chicks do have inborn expectations about the fact that intentional agents typically maintain a stable front–back orientation during locomotion. The distractor stimulus may, thus, appear to chicks as an agent that is “behaving in an odd way”, violating their expectations about the stability of body orientation. This could then stimulate further inspection of the “odd” agent by the chicks. Note that both the target and the distractor presented various features potentially associated with animacy perception (e.g. cues of self-propulsion such as movement against gravity, spontaneous changes in the motion direction and rotational movements, see Rosa-Salva et al. 2016, 2018). This explanation, however, would require an adjustment of our main underlying theory. As we mentioned above, it is typically assumed that social predispositions cause approach to the object that displays more cues of animacy or agency. Here, we would need to hypothesise that, rather than approaching a stimulus, because it fulfils the input conditions for an animate agent, chicks can also choose to approach to inspect a stimulus that violates some of their expectations about animate agents (which could be in line with the results of Lemaire et al. 2022). On the other hand, human adults and older children too have shown in some circumstances, a visual preference for a stimulus lacking some clear agentive traits (random movement of visual objects) compared to a chasing stimulus, which normally elicits strong agency/animacy perception (e.g. Rochat et al. 1997). In this study, adult participants declared that the random motion display “*was more interesting as it challenged participants’ propensity to detect invariant relations in the dynamics of the two discs*”.

A somewhat related interpretation has been proposed to account for a curious bias that domestic chicks present in their early foraging responses. Contrary to what has been shown when tested for their social predispositions, in foraging tasks, chicks show an unlearnt preference to peck at insect-like shapes that move along their shorter body axis. Indeed, chicks prefer to peck at these elongated targets when they move “sideways”, i.e. with their main body axis perpendicular to the motion trajectory (Clara et al. 2009). This was explained as a preference for preying on the “insects” whose movement direction violated chicks’ inborn expectations of the body orientation of potential prey. This could be adaptive by directing chicks’ preying behaviour towards injured or “abnormal” insects, whose reduced motility would make them easy prey.

If chicks’ sensitivity to agency cues can be expressed both through preferential responses towards target and distractor stimuli, for this hypothesis to acquire a predictive value, we would need to specify the factors that determine the switch between the two responses. It is, thus, crucial for future studies to define the circumstances under which this can happen. For example, does the tendency to inspect the “odd” stimulus depend on the presence or absence of other features of animacy? It could be hypothesised that, if two objects are both characterised by a sufficient animacy cues (and are, thus, both recognised as animate agents), chicks will decide to explore the one showing “unexpected behaviour”. However, similar phenomena were not observed in the paper of Rosa-Salva et al. (2018), despite the stimuli being very similar to the current ones. Why in this case chicks did not inspect the stimuli lacking parallelism, but rather preferred to approach the agents displaying that cue? Future works should investigate whether the responses to parallelism and to front–back consistency are systematically different in this regard.

It is worth mentioning that there is anecdotal evidence of chickens moving “backwards” in arousing situations (personal communication, Reviewer 2). This behaviour is also often attributed by farmers to diet-induced neurological abnormalities (e.g. nutritional Encephalomalacia, Boulianne et al. 2013). In this case, it has been reported that other flock members tend to display aggressive behaviour towards the backwards-walking individuals. These reports are not supported yet by empirical studies. However, if this behaviour is indeed part of this species’ ethogram, an agent moving back and forth might be particularly conspicuous to chicks. Future research should, thus, be devoted to systematically monitoring the presence of this behaviour in domestic chickens of different ages and the reaction of naïve individuals to simplified stimuli implementing these movement patterns.

Another interesting aspect of our data is that the preference for the agent with variable body orientation was largely confined to females. Differences in social behaviour between male and female chicks should be interpreted in light of the ecology of this species. In natural conditions, a feral cock controls a large territory, within which various females live (McBride et al. 1969). The dimorphic territorial behaviour of this species may, thus, promote sociality in females and aggressive or exploratory tendencies in males. Indeed, young chicks have long been known to display sex differences in their early social behaviour. For instance, female chicks remain in closer contact with the mother hen than males do (Workman and Andrew 1989) and are generally more motivated to reach social companions (Vallortigara et al. 1990, see also Vallortigara and Zanforlin 1988). Another notable sex difference is in the preference of young chicks for approaching familiar or novel objects after imprinting. It has been often reported that females display preferences for familiar social companions, while males are more attracted by (slightly) novel objects (e.g. Vallortigara and Andrew 1991; Vallortigara 1992; Regolin et al. 2000; Versace et al. 2017; see also Santolin et al. 2020). Males have also been known to be more aggressive, towards both familiar and unfamiliar social companions (Vallortigara 1992). Sex differences were also found when chicks were tested for their social predispositions for static face-like configurations that contained concentric eye-like features. Chicks were presented with an ambiguous stimulus that contained both a socially attractive feature (face-like configuration) and a fear-inducing feature (predator-like eyes). While normally chicks of both sexes are attracted by face-like stimuli (Rosa-Salva et al. 2010, 2011), in this case, females seemed to respond mainly to the social features of the stimulus, being more attracted by it (Rosa-Salva et al. 2012). On the contrary, males tended to avoid it, probably responding mostly to its predator-like features (Rosa-Salva et al. 2012; see also Adiletta et al. 2021). Sex differences have been reported in chicks’ responses to animate movement too. In a study by Miura and Matsushima (2012), naïve chicks of the Leghorn *Julia* strain were exposed to PLD, showing either biological motion (PLD of a walking hen) or various kinds of non-biological motion. After this priming experience, male chicks developed a preference for biological motion stimuli, independently of the animation to which they had been exposed. On the contrary, females showed a preference for the biological motion (walking hen) stimulus only after being exposed to it (while they failed to imprint on the other motion patterns). Moreover, only female chicks developed a selective preference for the PLD of the walking hen over that of a walking cat, after being primed by exposure to a random motion stimulus. These data suggest that, in males, exposure to any dynamic stimulus may elicit the subsequent expression of a general predisposition for biological motion. On the contrary, females seem to be endowed with more “specific” predispositions, which facilitate imprinting towards their species-specific walking pattern. Please note, however, that these effects may depend on the strain of chicks used (see Regolin et al. 2000 for a different pattern of sex-dimorphic behaviour in a similar task with chicks of the *Hybro* strain; see also Santolin et al. 2020, for evidence of strain-specific sex differences in chicks). Unfortunately, none of the studies reporting sex differences in social predispositions employed the same strain as the current study (Aviagen Ross 308), complicating the interpretation of our results.

To sum up, the literature reports multiple instances of sex differences in chicks’ early social behaviour, including their predispositions for animate motion. However, such sex differences are not always present, even when similar tasks are employed (e.g. see Rosa-Salva et al. 2010 vs. Rosa-Salva et al. 2012; Adiletta et al. 2021; Rosa-Salva et al. 2016, 2018 vs. the current study). The situation is further complicated by the use of different strains of chicks, which seem to diverge in the presence or type of sex dimorphism (Miura and Matsushima 2012; Regolin et al. 2000; Santolin et al. 2020). Among other things, females can be more motivated to social reinstatement (Vallortigara et al. 1990) and more likely to interpret ambiguous stimuli as social companions (Rosa-Salva et al. 2012). Moreover, female chicks might also be endowed with more “specific” predispositions, which facilitate imprinting towards species-specific biological motion patterns (Miura and Matsushima 2012). The fact that, in the current study, the preference for the agent with inconsistent body orientation was limited to females, is in line with those traits.

In conclusion, we want to reiterate that the experiments reported here did not find evidence in support of the original hypothesis that visually naïve chicks will have a social predisposition towards objects displaying front–back consistency during motion (i.e. consistency in which end is leading and which is trailing). We also did not find evidence in support of the interim post hoc hypothesis that our stimuli elicited a social predisposition for gait-like biological motion. The reported results document, in visually naïve female chicks, an unpredicted behavioural preference for objects whose movement exhibits numerous characteristics of animate motion, yet lacks consistency in which of its ends is leading and which is trailing. We speculated that this could indicate a preference for agents showing higher behavioural variability, or a tendency to explore animate agents performing “odd behaviours”. We also discussed possible reasons for the effect being limited to females only. While the reported effect still demands an explanation, we put forward that it may be revealing of previously unrecognised complexity in chick’s innate responses to animate motion.

## Supplementary Information

Below is the link to the electronic supplementary material.

Supplementary file1 (MP4 7716 KB)

Supplementary file2 (MP4 6830 KB)

Supplementary file3 (XLSX 11 KB)

## Data Availability

The full dataset used for this study is available as supplementary material S3.
